# Molecular analysis of polymorphic species of the genus *Marshallagia* (Nematoda: Ostertagiinae)

**DOI:** 10.1186/s13071-020-04265-1

**Published:** 2020-08-12

**Authors:** Abdurakhim Kuchboev, Khanifakhon Sobirova, Rokhatoy Karimova, Oybek Amirov, Georg von Samson-Himmelstjerna, Jürgen Krücken

**Affiliations:** 1 Institute of Zoology, Uzbekistan Academy of Sciences, Bogishamol str. 232B, Tashkent, 100053 Uzbekistan; 2 Institute for Parasitology and Tropical Veterinary Medicine, Freie Universität Berlin, Robert-von-Ostertag-Str. 7–13, 14163 Berlin, Germany

**Keywords:** *Marshallagia*, Polymorphic species, Parasitic nematodes, Ruminants, Barcoding gap

## Abstract

**Background:**

The genus *Marshallagia* (Family Haemonchidae, subfamily Ostertagiinae) contains multiple species of nematodes parasitising the abomasum (or duodenum) of ruminants, in particular of Caprinae. Male specimens have been described to be polymorphic with the frequent/major morphotype initially described in the genus *Marshallagia* while the minor/rare morphotype was initially often placed in the genus *Grossospicularia*. Due to common morphological features, certain pairs of morphotypes were suggested to belong to the same species such as *Marshallagia marshalli*/*M. occidentalis*. However, molecular evidence to confirm these pairs of morphotypes belonging to the same species is missing.

**Methods:**

In the present study, *Marshallagia* sp. were collected from domestic sheep in Uzbekistan. Male specimens were morphologically described with particular emphasis on the structure of the bursa copulatrix. After DNA isolation from morphologically identified specimens, PCRs targeting the ribosomal internal transcribed spacer 2 (ITS2) and mitochondrial cytochrome *c* oxidase subunit 1 (*cox*1) regions were conducted. After Sanger sequencing, maximum likelihood phylogenetic analyses and pairwise identities between sequences were calculated.

**Results:**

The major morphotypes of *M. marshalli*, *M. schumakovitschi* and *M. uzbekistanica* and the minor morphotypes *M. occidentalis*, *M. trifida* and *M. sogdiana* were identified and their morphology was documented in detail. ITS2 sequences showed little variation and did not allow diagnosing species. In contrast, phylogenetic analysis of *cox*1 sequences identified highly supported clusters and verified that *M. marshalli*, *M. occidentalis* and *M. uzbekistanica* are different morphotypes of the species *M. marshalli* while *M. schumakovitschi* and *M. trifida* represent distinct morphotypes of *M. trifida*. For *M. sogdiana* no corresponding major morphotype could be identified in the present study. Due to a large barcoding gap, comparison of *cox*1 sequences in terms of percent identity was sufficient to reliably assign the sequences to a particular species without phylogenetic analysis.

**Conclusions:**

The data presented here create a framework that will allow the classification of other members of the genus in the future and underline that parallel morphological and molecular analysis of specimens is crucial to improve the taxonomy of polymorphic species.
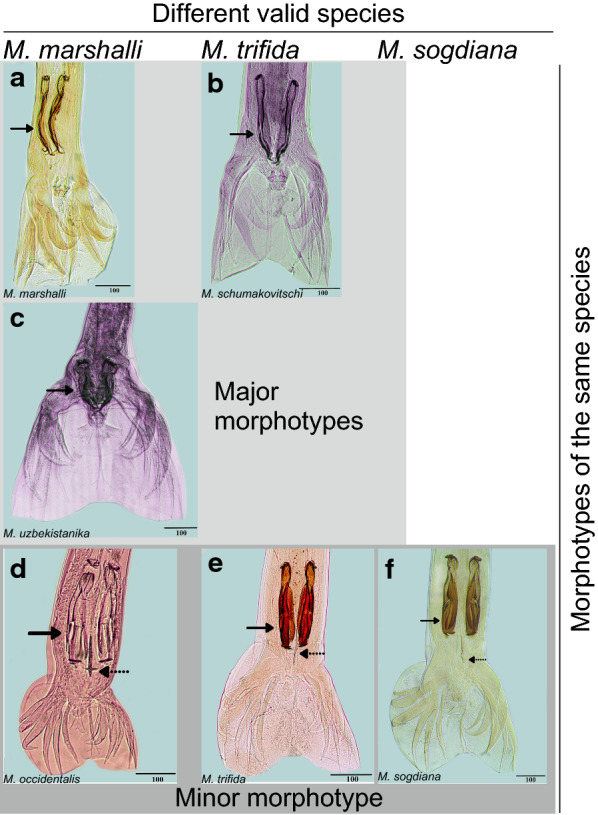

## Background

Nematodes of the genus *Marshallagia* Orloff, 1933 are parasites of the abomasum and duodenum of free-ranging and domesticated ruminants and are most often associated with Caprinae from the Holarctic region [1, 2]. They have a considerable impact on the metabolism of host animals frequently leading to economic losses [3–5]. According to our data, the species of *Marshallagia marshalli* Ransom, 1907 is widespread in Uzbekistan and it is the most frequently detected member of this genus [6].

Drozdz [7, 8] described that morphological dimorphism is common among males of several species of the subfamily Ostertagiinae, i.e. presence of “major” and “minor” morphotypes in the same host. Major morphotypes are named due to their higher frequency relative to minor morphotypes, not due to differences in size. In contrast to males, females are monomorphic, and this situation has often led to the description of distinct species for these male morphotypes.

In the genus *Marshallagia*, five dimorphic species have been described by Drozdz [9]. Later, 12 dimorphic species of *Marshallagia* were differentiated by morphological descriptions incorporating the synlophe in males, the structure of the spicules as well as the genital cone in major and minor morphotype males [2]. In particular, structural characters of the spicules and genital cone were used to distinguish between major and minor morphotypes. The major morphotypes were usually placed in the genus *Marshallagia* while minor morphotypes were often allocated to *Grosspiculagia* Orloff, 1933. Thus, major and minor morphotypes of the same species have historically been described as different nominal species, often in separate genera [9, 10]. However, there are also morphological features that are shared between major and minor morphotypes but can be used to discriminate between species including in particular details of the morphology of esophagus and synlophe as well as the shape of the rays of the copulatory bursa [9]. For instance, *M. occidentalis* Ransom 1907 (minor morphotype) has clearly distinguishable morphological features discriminating it from *M. marshalli* (major morphotype), but there are clear hints that *M. marshalli* and *M. occidentalis* are synonyms and represent only different morphotypes of *M. marshalli*, whereas *M. mongolica* Shumakovitschi, 1950 appears to be a major morphotype while *M. grossospiculum* Li, Yin, Kong & Jang, 1987, which corresponds to *Marshallagia* sp. 1 of Drozdz [9], is most likely a minor morphotype of the same species. The same relationship presumably also applies to *M. schumakovitschi* Kadyrov, 1959 (major morpotype) and *M. trifida* (Guille, Marotel & Penisset, 1911) (= *Marshallagia* sp. 2 of Drozdz [9]) (minor morphotype) [2].

Even less is known about the occurrence of cryptic species in other frequently occurring representatives of the Ostertagiinae, especially in *M. marshalli* due to its presence in several host species [2, 11]. Regarding nomenclature, taxonomy and phylogeny of the genus *Marshallagia*, Hoberg et al. [2] proposed that additional studies are required combining morphological and molecular analysis from individual specimens to confirm descriptions of the major species *M. skrjabini* as well as the minor species *M. belockani* and *M. sogdiana*. Moreover, it remains an open question as to whether or not *M. marshalli*/*M. occidentalis* is distributed in North America and in Eurasia. *Marshallagia marshalli*/*M. occidentalis* is the only species of the genus with a Holarctic distribution but detailed comparisons between North American and Central Eurasian populations have not been conducted. Therefore, it should be considered that there are two closely related species with more restricted geographic distribution patterns [2] and that reports of identification of *M. marshalli* from Eurasian ruminants are due to misidentification.

Based on studies by Drozdz [9], Hoberg et al. [2] and Wyrobisz et al. [12] listed five polymorphic species in the genus *Marshallagia*: *M. marshalli/M. occidentalis*; *M. lichtenfelsi/M. lichtenfelsi* f. minor Hoberg Abrams, Pilit & Jenkins, 2012; *M. mongolica/M. grossospiculum*; *M. schumakovitschi/M. trifida*; and *M. skrjabini/M.belockani*. However, to date no consensus on the species composition of the genus *Marshallagia* has been obtained.

Within the Strongylida, species distinction supported merely by morphological features is difficult, and requires confirmation by means of molecular methods. For instance, recent data have suggested that *Cooperia spatulata* is just a morphotype of *C. punctata* [13] while there is evidence that the small strongyle morphospecies *Cylicostephanus calicatus* and *C. minutus* are in fact cryptic species complexes of at least two and three genospecies, respectively [14]. The taxonomy of Ostertagiinae is mainly complicated by complex relationship between species and morphotypes in the genus *Teladorsagia*, but complexity may also be expected among other Ostertagiinae (e.g. in the genera *Ostertagia* and *Marshallagia*) [12]. Studies devoted to the problems of taxonomy of strongylid nematodes have shown that non-coding regions of rRNA genes, in ITS1 and particularly ITS2, are well suited to allow taxonomic discrimination and this also applies to members of the Ostertagiinae [15–24]. These studies have led to important insights in the evolution and solved general questions of phylogeny of Ostertagiinae including confirmation of conspecificity for *M. marshalli* and *M. occidentalis* [25]. Furthermore, questions regarding conspecificity of supposed major and minor morphs and cryptic species within the genus of *Orloffia* Drozdz, 1965 were solved [26]. There is no unanimous opinion about the taxonomic independence and specific composition of the genus *Orloffia*. At the same time, there are several species and genera, for which important taxonomic and phylogenetic questions remain unresolved. Additional molecular analyses of ribosomal and mitochondrial DNA will allow understanding these problems in more depth.

Regarding species identification in terms of barcoding properties, mitochondrial DNA sequences were shown to be superior to ribosomal spacers [27]. Recent work on *Cooperia* spp. (Cooperiidae, Cooperinae) and *C. minutus* (Strongylidae, Cyathostominae) revealed that combined analyses of mitochondrial and nuclear marker sequences improved species identification and phylogenetic analyses [13, 14].

This study aimed to provide sequence data on mitochondrial cytochrome *c* oxidase subunit 1 (*cox*1) and nuclear ribosomal intergenic spacer 2 (ITS2) DNA for some species or morphotypes of the genus of *Marshallagia.* The objective was to morphologically identify individual *Marshallagia* sp. specimens from Uzbekistan to the species and morphotype level followed by obtaining molecular ITS2 and *cox*1 data from the same specimens in order to clarify the taxonomic status of the local *Marshallagia* species/morphotypes.

## Methods

### Parasite collection and examination

All parasite material was collected at necropsy from domestic sheep (*Ovis aries*) from farms in the Kitob district (Kashkadarya region) and in the Shofirkon district (Bukhara region) in Uzbekistan. Mature *M. marshalli* and *M. occidentalis* worms were collected from the mucosa of the abomasum of domestic sheep (*Ovis aries*) in Kashkadarya region (June 2016) while *M. schumakovitschi*, *M. trifida*, *M. sogdiana* and *M. uzbekistanica* were collected from abomasum of sheep in Bukhara region (July 2016). Male specimens were manually cut in two parts: the posterior region was cleared in phenol alcohol (80% melted phenol and 20% ethanol) for examination of the morphological features and the anterior and middle parts were fixed in 70% ethanol for the molecular studies (Table 1).

**Table 1:** Specimens of *Marshallagia* species collected from hosts in Uzbekistan and additional paratype males from the Central Helminthological Museum (Moscow, Russia) for morphological analyses

Parasite species	Locality	No. of specimens	Collection
*Marshallagia marshalli*	Kashkadarya district, Uzbekistan	26 ♂	CPIZ 10270^a^
*M. occidentalis*	Kashkadarya district	22 ♂	CPIZ 10278
*M. schumakovitschi*	Bukhara district, Uzbekistan	17 ♂	CPIZ 10271
*M. trifida*	Bukhara district	18 ♂	CPIZ 10291
*M. sogdiana*	Bukhara district	12 ♂	CPIZ 10293
*M. uzbekistanica*	Bukhara district	5 ♂	CPIZ 10292
*Marshallagia marshalli*	Volgograd district, Russia	2 ♂	CHM 14768^b^
*M. schumakovitschi*	Osh district, Kirgizstan	2 ♂	CHM 22289

^a^CPIZ - Collection of the Parasitology Institute of Zoology Uzbekistan Academy of Sciences, Uzbekistan

^b^CHM - Central Helminthological Museum Russian Institute of Parasitology, Animal and Plant named after K.I. Skrjabin, Russia

### Morphological identification

*Marshallagia* species were identified according to morphological and morphometrical characters using literature data [1, 2, 9]. All adult male worms isolated from each sheep were morphologically analyzed to identify parasite species. The species identification was established based on caudal bursa according to the features proposed by above literature, especially morphological characters and measurements of spicules, dorsal ray and gubernaculum (Table 1).

An equal mixture of lactic acid and glycerin was used to enlightenment the posterior part of the studied nematodes without additional staining. Also included in the present paper are morphological studies made by the authors analyzing specimens (paratypes or sintypes) of species (*M. marshalli* Ransom, 1907 and *M. schumakovitschi* Kadyrov, 1959) in the Central Helminthological Museum FGBNU, Russian Institute of Parasitology, Animal and Plant named after K. I. Skrjabin, Moscow. For this purpose, a microscope ML 2000 equipped with a digital camera (Meiji, Saitama, Japan) was used.

### DNA extraction

For DNA isolation, at least a single specimen of each species/morphotype was used. Before isolation of genomic DNA, the ethanol was removed and the adult nematodes were washed with sterile water and DNA was extracted using the NucleoSpin^®^ Tissue Kit (Macherey-Nagel, Düren, Germany) in accordance with the manufacturer’s protocol. The DNA was eluted with 50 µl elution buffer provided in the kit and stored at − 20 °C until further use. The extracted DNA was quantified on a Take3 plate in an Epoch plate reader (Biotek, Berlin, Germany).

### PCR and cloning

PCRs were conducted using (i) a combination of the forward and reverse primers flanking the complete ITS2 region [28] and (ii) a partial *cox*1 gene fragment [29] (Table 2). PCR reactions contained 0.2 mM dNTPs, 250 nM of each primer, 0.4 U Phusion Hot Start II High-Fidelity DNA polymerase (Thermo Fisher Scientific, Darmstadt, Germany) and 2 μl template DNA in 20 μl 1× HF buffer. PCRs were performed on a C1000 or S1000 PCR cycler (Bio-Rad, Feldkirchen, Germany). PCR products were purified using DNA Clean & Concentrator^TM^-5 (Zymo Research, Freiburg, Germany) and amplification products were analyzed by agarose gel electrophoresis in 1.0–1.5% agarose gels. Purified fragments were ligated into the StrataClone Blunt PCR Cloning Vector pSC-B-amp/kan (Agilent, Waldbronn, Germany) and transformed into StrataClone SoloPack competent *Escherichia coli* cells according to the manufacturer’s protocol. Plasmid DNA was purified using the EasyPrep1 Pro kit (Biozym, Hessisch Oldendorf, Germany) and sent for sequencing to LGC Genomics (Berlin, Germany).

**Table 2:** Primers and PCR conditions used for molecular analyses of nematodes

Primer	Sequence (5′–3′)	Initial denaturation	Denaturation/annealing/extension	Final elongation
NC1	ACGTCTGGTTCAGGGTTGTT	98 °C for 30 s	40×: 98 °C for 10 s; 55 °C for 30 s; 72 °C for 30 s	72 °C for 10 min
NC2	TTAGTTTCTTTTCCTCCGCT			
COI_Nema_Fw	GAAAGTTCTAATCATAARGATATTGG	95 °C for 2 min	35×: 95 °C for 1 min; 48 °C for 1 min; 72 °C for 1 min	72 °C for 5 min
COI_Nema_Rv	ACCTCAGGATGACCAAAAAAYCAA			

### Sequence comparisons and phylogenetic analyses

Sequences from the present study were analyzed together with sequences previously deposited in GenBank. As an outgroup, two sequences per gene from the species *Teladorsagia circumcincta,* were included. Accession numbers of all sequences from specimens investigated in the present study are provided in Table 3. The ITS2 and *cox*1 sequences were aligned using MAFFT (multiple sequence alignment using fast Fourier transformation) in the Q-INS-I modus that takes predicted RNA secondary structures into account [30] and the M-COFFEE modus of T-Coffee (Tree-based Consistency Objective Function for alignment Evaluation) [31], respectively. The *cox*1 alignment was manually edited to ensure that codons were not interrupted by gaps. For calculation of relative identity (%) between sequences, alignments were analyzed using the dist.dna function in the *ape 4.0* (Analyses of Phylogenetics and Evolution) package [32] in R 4.0.0 statistics software [33]. Identities were calculated as “raw” identities and pairwise deletion of positions with gaps was turned on. Comparisons of sequences within the genus *Marshallagia* were sorted into the individual intraspecies comparisons and a single category containing all interspecies comparisons. The identity in percent for all these comparisons within the genus *Marshallagia* were compared using the Kruskall-Wallis test followed by a Conover-Iman *post-hoc* test with the function posthoc.kruskal.conover.test as implemented in the R package *PMCMR 4.3* [34]. All *P*-values below 0.05 were considered to be statistically significant. Scatter plots were visualized using GraphPad Prism 5.03 (GraphPad, La Jolla, USA).

**Table 3:** Cytochrome *c* oxidase subunit 1 (*cox*1) and second internal transcribed spacer (ITS2) sequences of *Marshallagia* species from Uzbekistan and the GenBank database from different geographical origins used in this study

Species	Voucher	GenBank ID	
		*cox*1	ITS2
*Marshallagia marshalli*	M2	MT116991	MT110920
*M. marshalli*	M12	MT116992	MT110919
*M. occidentalis*	M14	MT116997	MT110967
*M. schumakovitschi*	M3	MT116993	MT110926
*M. schumakovitschi*	M6	MT116994	MT110928
*M. schumakovitschi*	M8	MT116995	MT110929
*M. schumakovitschi*	M10	MT116996	MT110927
*M. trifida*	M5	MT116998	MT118027
*M. trifida*	M9	MT116999	MT118028
*M. sogdiana*	M4	MT117000	MT118024
*M. sogdiana*	M7	MT117001	MT118025
*M. sogdiana*	M13	MT117002	MT118026
*M. uzbekistanica*	M1	MT116990	MT118029

Phylogenetic analyses were conducted on a single gene level. First, substitution saturation tests were conducted according to Xia et al. [35] using DAMBE 5 (Data Analysis in Molecular Biology and Evolution) software [36]. DAMBE 5 was also used to split the *cox*1 alignment into one partition for the first and second and another partition for the third codon position. Maximum likelihood phylogenetic trees were calculated using IQ-TREE [14] on the IQ-TREE server (http://iqtree.cibiv.univie.ac.at). Using the ModelFinder option of IQ-TREE [37], auto-determination of the best model applying the Bayesian information criterion was performed including models with FreeRate heterogeneity. Ultrafast bootstrapping (1000 bootstrapped replicates) [38] and the Shimodaira-Hasegawa approximate likelihood ratio test (SH-aLRT) (1000 replicates) [39] were used to obtain node support statistics. The command line in IQ-TREE for ITS2 sequences was: iqtree -s infile.fas -st DNA -m TESTNEW -bb 1000 -alrt 1000. For *cox*1 sequences, separate models were fitted for codon positions 1 and 2 *vs* codon position 3 using the command line: iqtree -s COI_FcC_infile.fas -spp partition_file.txt -pre infile.fas –m TESTNEW -bb 1000 -alrt 1000. Phylogenetic trees were visualized in FigTree 1.1.4 and further edited in CorelDraw 20.

## Results and discussion

### Morphological identification

**Fig. 1:**
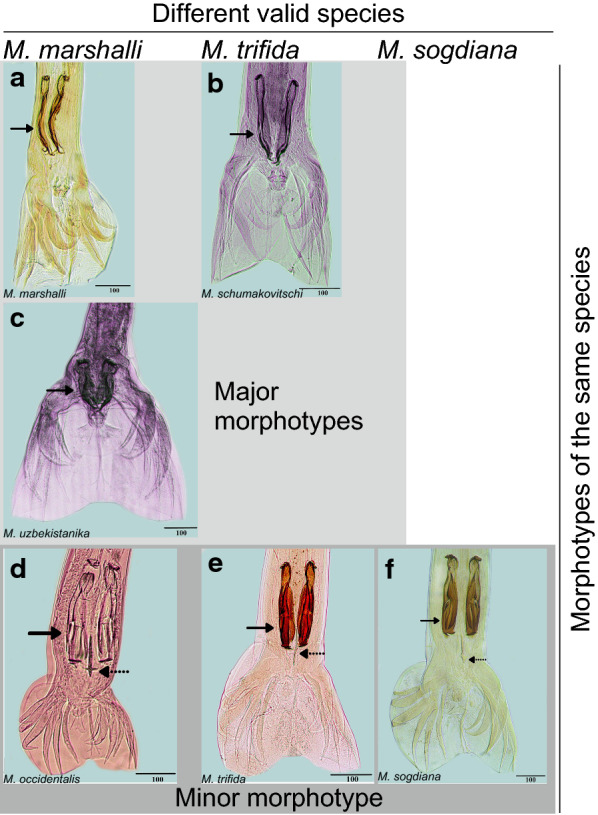
*Marshallagia* spp. n. f. major and minor males showing ventral view of primary structural characters. The spicules and the gubernaculum are indicated by arrows and dotted arrows, respectively). Scale-bars are given in micrometers. **a** *M. marshalli*; **b** *M. schumakovitschi*; **c** *M. uzbekistanica*; **d** *M. occidentalis*; **e** *M. trifida*; **f** *M. sogdiana*

Six morphotypes of *Marshallagia* species were found within the present material. Major and minor *Marshallagia* species were isolated from domesticated sheep in Uzbekistan, separated according to their morphological identification (Fig. 1) and morphometric comparison (Table 4) and assigned to *M. marshalli*, *M. schumakovitschi*, *M. uzbekistanica*, *M. occidentalis*, *M. trifida* and *M. sogdiana*. As detailed above, Drozdz [7, 8] described the phenomenon of regular co-occurrence of rather rare (minor) species in pairs with the more numerous (major) species, which, together with subtle morphological features, lead to the hypothesis that they represent different morphotypes of the same species. Based on this hypothesis, the analyzed specimens of *Marshallagia* were divided into six separate morphotypes that were grouped into three species with co-existing morphotypes (Fig. 1). The original micrographs of the specimens that were used for molecular analysis are presented for one exemplary individual per morphotype: *M. marshalli* (Fig. 1a); *M. schumakovitschi* (Fig. 1b); *M. uzbekistanica* (Fig. 1c); *M. occidentalis* (Fig. 1d); *M. trifida* (Fig. 1e); and *M. sogdiana* (Fig. 1f). A detailed morphological description of the different morphotypes is given in Additional file 1: Text S1.

**Table 4:** Morphometric data for the male specimens (major and minor morphotypes) of the morphospecies of *Marshallagia* in domestic sheep from Uzbekistan, based on new observations during the current study

Characters	*M. marshalli* (*n* = 26)	*M. occidentalis* (*n* = 22)	*M. schumakovitschi* (*n* = 17)	*M. trifida* (*n* = 18)	*M. sogdiana* (*n* = 12)	*M. uzbekistanica* (*n* = 5)
Body length	680–1400 (1050 ± 39)	1000–1600 (1330 ± 51)	1140–1380 (1190 ± 73)	830–17000 (1396 ± 88)	940–1850 (1355 ± 177)	734–1586 (1240 ± 28)
Body width	125–257 (207 ± 9)	180–260 (210 ± 8)	112–201 (175 ± 4)	131–212 (170 ± 9)	129–235 (169 ± 21)	294–428 (340 ± 8)
Diameter of anterior region	11–26 (21 ± 1)	36–46 (41 ± 1)	21–33 (26 ± 3)	41–62 (50 ± 3)	39–65 (47 ± 4)	17–23 (21 ± 4)
Distance from cervical capsule to anterior extremity	292–491 (381 ± 13)	371–479 (414 ± 8)	391–455 (405 ± 15)	337–581 (424 ± 28)	165–215 (188 ± 9)	401–448 (429 ± 10)
Distance from nerve-ring to anterior extremity	239–391 (318 ± 7)	291–416 (350 ± 10)	220–388 (290 ± 12)	251–411 (320 ± 10)	247–395 (295 ± 12)	151–221 (173 ± 9)
Esophagus length	211–319 (242 ± 18)	751–991 (890 ± 20)	781–957 (860 ± 20)	672–932 (774 ± 31)	719–793 (748 ± 23)	65–96 (78 ± 9)
Esophagus width	26–39 (33 ± 3)	62–93 (71 ± 5)	58–87 (65 ± 4)	59–72 (62 ± 3)	53–68 (59 ± 4)	63–74 (68 ± 4)
Spicule length	211–311 (250 ± 10)	218–373 (280 ± 9)	218–301 (240 ± 10)	241–391 (295 ± 9)	254–383 (284 ± 15)	102–147 (124 ± 7)
Spicule width	42–54 (48 ± 3)	52–61 (57 ± 2)	53–59 (53 ± 3)	43–57 (49 ± 4)	47–51 (48 ± 5)	38–46 (41 ± 3)
Dorsal ray length	321–483 (370 ± 11)	139–317 (200 ± 20)	251–375 (300 ± 10)	231–338 (259 ± 11)	223–315 (253 ± 11)	223–378 (241 ± 11)
Bursa length	285–452 (360 ± 8)	653–746 (690 ± 8)	311–415 (357 ± 10)	611–884 (709 ± 12)	593–875 (745 ± 12)	278–548 (366 ± 13)

The most reliable characters for differentiation among species of *Marshallagia*, and specifically of the major morphotypes of the respective species, include the placement of the trifurcation of the spicule tips, the form of the dorsal and ventral processes (relative length, curved or straight), and the chitinized structure of the tip of respective processes.

The three major morphotypes designated as *M. marshalli*, *M. schumakovitschi* and *M. uzbekistanica* (Fig. 1a-c) differ by the distal ends of spicule processes, strongly curved for the first one and with tubercles for the second and third species. All three major morphotypes are characterized by the absence of a gubernaculum (Fig. 1a–c).

*Marshallagia marshalli* (Ransom, 1907) is the type-species for the genus. Ransom [40, 41] described this species and the minor morphotype, *M. occidentalis*, based on specimens in domesticated sheep (*O. aries* L.) from North America. In specimens of the major morphotype of *M. marshalli* the spicules are strongly curved in lateral view, 210–310 µm in length, 42–54 µm in width; eyelet at trifurcation prominent; with dorsal and ventral process nearly equal in length; gubernaculum absent or strongly chitinized (Fig. 1a). The *M. marshalli* morphotypes studied by us corresponded to the morphology of the paratype of *M. marshalli* (CHM 14768) (Table 1).

*Marshallagia schumakovitschi* differs from *M. marshalli* in the structure of the spicule. The most distal sixth part of the spicules is divided into three processes. Dorsal and ventral processes are nearly equal in length, the ventral process terminates in a simple tip, which may be bent; the dorsal process is weakly sclerotized, blunt and not strongly recurved and extends to near the termination of the main shaft (Fig. 1b).

*Marshallagia uzbekistanica* is a morphologically atypical form placed in the genus *Marshallagia* in the original description. Spicules are asymmetric (Fig. 1c). The characteristic of *M. uzbekistanica* turned out to be a peculiar structure of the spicules, which are weakly chitinized at the proximal end and granular in structure [42].

Minor morphs represented in the present study by the morphotypes *M. occidentalis*, *M. trifida* and *M. sogdiana* correspond to the diagnosis of the genus *Grosspiculagia*, which is now considered to be a synonym of *Marshallagia*. In contrast to the major morphs, all minor morphs have thick spicules that are split into three processes. The two more massive processes have cap-shaped distal ends and sometimes a hook-like outgrowth. In contrast to the major morphs, all minor morphs have a transparent, sometimes subtle gubernaculum (Fig. 1d–f).

*Marshallagia occidentalis* represents the minor morphotype of *M. marshalli* [9]. Near the middle of the spicule length, spicules are divided into three processes: two ventral and one dorsal. A gubernaculum is present but to the rear its diameter is strongly reduced (Fig. 1d).

*Marshallagia trifida* is the minor morphotype of *M. schumakovitschi* and can be identified based on its spicule structure [2, 9]. The ventral process of the spicules is strongly curved; the dorsal process extends to the tip of the main shaft of the spicule. The gubernaculum is fusiform (Fig. 1e).

*Marshallagia sogdiana* is the minor morphotype of *M. skrjabini* Asadov, 1954 and was transferred to that species as a new combination of morphs [2]. The proximal ends of the spicules are characterized by the presence of a peculiar, disc-like structure (Fig. 1f). In the middle of the spicule it is divided into three processes: two ventral and one dorsal. The distal spicules have a membrane in the form of a sheath. A gubernaculum is present.

Thus, based on morphological characters and morphometric comparison of males of *Marshallagia* sp., two pairs of major and minor morphotypes were identified: *M. marshalli*/*M. occidentalis*, *M. schumakovitschi*/*M. trifida* while for the pair *M. skrjabini/M. sogdiana* only the minor morphotype was found (Table 4). In addition, *M. uzbekistanica* was identified as an unusual major morphotype. The data on the morphology of these species from samples collected in Uzbekistan presented here closely corresponds to the previously published data by Asadov [43], Ivashkin [1] and Hoberg et al. [2]. Herein, to scrutinize the identity of the morphs of the genus *Marshallagia*, molecular studies were conducted in particular to confirm which major and minor morphotypes belong to the same species.

### Molecular analyses

For adult worms collected from domesticated sheep from Uzbekistan and morphologically identified as *M. marshalli*/*M. occidentalis*, *M. schumakovitschi*/*M. trifida*, *M.sogdiana* and *M. uzbekistanica*, PCR products for the nuclear ITS2 rRNA (321–325 bp excluding the primers) and mitochondrial partial *cox*1 (696 bp) genes were amplified, cloned and sequenced. All sequences were deposited in GenBank under the accession numbers provided in Table 3.

### Internal transcribed spacer 2

Identity between all ITS2 sequences of the genus *Marshalagia* from the present study (*n* = 13) or from GenBank revealed between 89.7% and 100% identity with 85% of the pairwise comparisons showing > 95% identity. A maximum likelihood phylogenetic tree calculated from ITS2 sequences from the present study plus those available in GenBank using two representative *T. circumcincta* sequences as an outgroup is shown in Additional file 2: Figure S1. The phylogram reveals that the ITS2 sequence contains virtually no phylogenetic signal since (i) there are barely any clusters of sequences showing high statistical support, (ii) sequences assumed to come from different species were virtually identical and (iii) the sequences assigned to the same species are found scattered all over the tree. This indicates that ITS2 sequences are not suitable to address taxonomic or even phylogenetic questions within the genus *Marshallagia.* This is in agreement with other recent studies analyzing closely related strongyle nematodes showing that ITS2 is an excellent marker to identify the genus, but that closely related species differ only minimally in their ITS2 sequence and that the phylogenetic signal obtained from ITS2 sequences of *Marshallagia* specimen was not reliable [13, 14].

### Cytochrome *c* oxidase subunit 1 gene

**Fig. 2:**
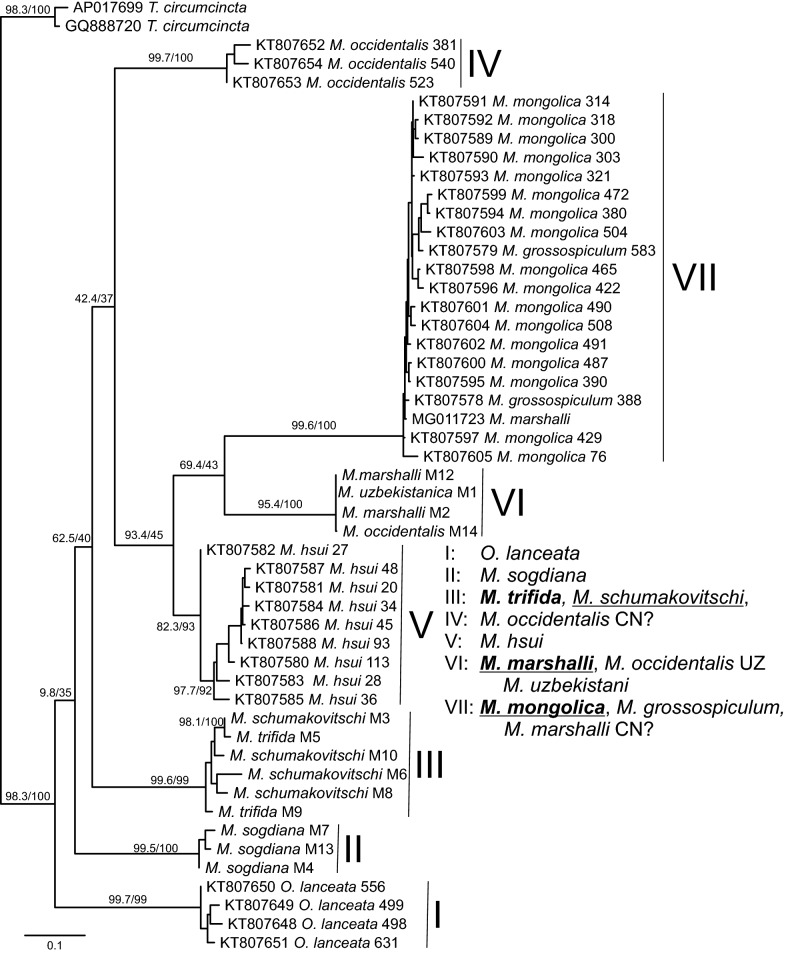
Maximum likelihood phylogentic tree for *Marshallagia* species based on cytochrome *c* oxidase subunit 1 (*cox*1) gene. *Teladorsagia circumcincta* was used as the outgroup while *Ostertagia lanceata* was included since data are annotated as *Marshallagia lanceata* in GenBank. Branch support is presented with results of the rapid bootstrap analysis before and of the Shimodaira-Hasegawa approximate likelihood ratio test behind the slash. Seven highly supported clusters (indicated by Roman numbers I-VII from basal to distal) are considered valid species: *O. lanceatea* (I); *M. sogdiana* (II); *M. trifida* (III) including the morphotype *M. schumakovitschi*; the presumably misidentified *M. occidentalis* from China (IV); *M. hsui* (V); *M. marshalli* (VI) including the morphotypes *M. occidentalis* and *M. uzbekistanica* from Uzbekistan; and *M. mongolica* (VII) including the morphotype *M. grossospiculum* and a presumably misidentified *M. marshalli* from China. If more than one morphotype is present in a cluster, the valid species name is printed in bold. Numbers after species names show voucher designations from Lv et al. [44] while M1–M14 are voucher designations from the present study. *Abbreviations*: CN, China; UZ, Uzbekistan; ?, apparently misidentified specimens

The phylogenetic tree calculated from *cox*1 sequences from the present study or downloaded from GenBank identified seven highly supported clusters (named I-VII from basal to distal operational taxonomic units (OTUs) in Fig. 2) with very low variability within the clusters. All available *Marshallagia* sequences come from only three studies. One from China [44] reporting 36 *cox*1 sequences, another Chinese study reporting a complete mitochondrial genome annotated as *M. marshalli* [45] and 13 sequences from the present study. The clusters I (*Ostertagia lanceata* erroneously assigned to the genus *Marshallagia* by Lv et al. [44] as indicated by Hoberg et al. [2], IV (*M. occidentalis*) and V (*M. hsui*) contained only sequences reported by Lv et al. [44]. In contrast, clusters II (*M. sogdiana*), III (*M. schumakovitschi* and *M. trifida*) and V (*M. marshalli*, *M. occidentalis* and *M. uzbekistanica*) contained only sequences from the present study. Only in cluster VII (*M. mongolica*, *M. grossospiculum* and *M. marshalli*) sequences from two studies are mixed, i.e. 17 *M. mongolica* and two *M. grossospiculum* sequences published by Lv et al. [44] and the single complete mitochondrial genome assigned to *M. marshalli* reported by Sun et al. [45].

The fact that two species sequences (*M. marshalli* and *M. occidentalis*) both occur in two different clusters clearly indicates that at least some of the published specimens were morphologically misidentified. Neither Lv et al. [44] nor Sun et al. [45] provided any morphological data for the specimens used to obtain their DNA sequences. Lv et al. [44] not even mentioned the criteria used for identification or any species identification key while Sun et al. [45] explicitly stated that morphological identification was difficult, and that molecular identification was used. They report that the ITS sequences obtained from their specimens were 99% identical to sequences KT428384 and HQ389231 deposited in GenBank as *M. marshalli*. However, in the ITS2 phylogenetic analysis presented in Additional file 2: Figure S1, these two sequences are (i) not grouped closely together and (ii) also virtually identical to ITS2 sequences deposited in GenBank for other *Marshallagia* species. Based on the present findings it can be assumed that the complete mitochondrial genome reported by Sun et al. [45] does not represent a *M. marshalli* but instead a *M. mongolica* sequence.

**Fig. 3:**
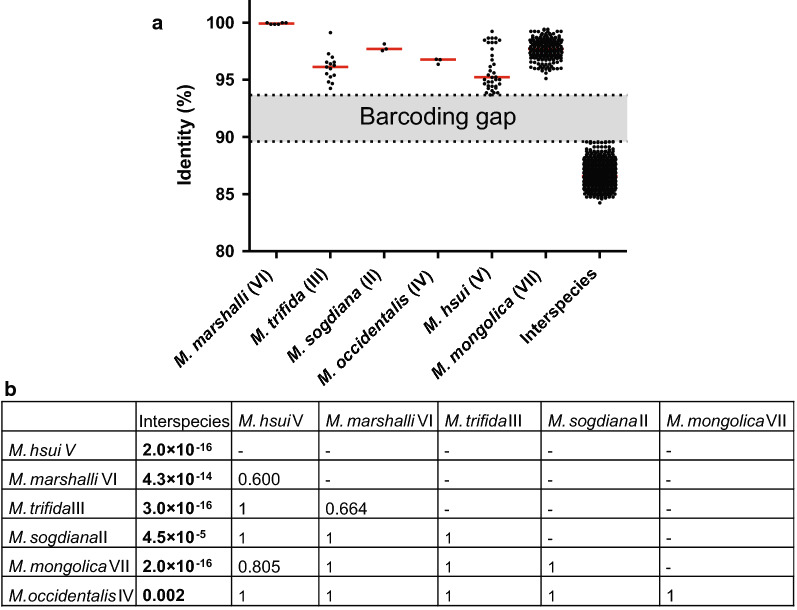
Sequence identity for cytochrome *c* oxidase subunit 1 (*cox*1) gene comparisons within and between species. The species *Marshallagia sogdiana* (II), *M. trifida* (III) including the morphotype *M. schumakovitschi*, the presumably misidentified *M. occidentalis* from China (IV), *M. hsui* (V), *M. marshalli* (VI) including the morphotypes *M. occidentalis* and *M. uzbekistanica* and *M. mongolica* including the morphotype *M. grossospiculum* were included. The different groups (II-VII) correspond to the groups in the phylogenetic tree in Fig. 2. **a** Scatterplots with medians are shown for all comparisons within these particular species or for any inter-species comparison. The barcoding gap between intra- and interspecies comparisons is indicated by the gray area. Median values for comparisons were significantly different in a Kruskal-Wallis H test (*χ*^2^ = 877.95, *df* = 266, *P* < 0.001). **b** A Conover *post-hoc* test comparing all different groups revealed that identities of all intra-species comparisons were significantly higher than interspecies comparisons. In contrast, identities of the intra-species comparisons for different species did not show any significant difference

Considering each of the highly supported major clusters in Fig. 2, identification of a valid species can be further confirmed by looking at the barcoding gap. Figure 3 shows raw percent identity for all pairwise comparisons between *Marshallagia* sp. sequences from the alignment used to calculate the *cox*1 phylogenetic tree in Fig. 2. It clearly exhibits that all intraspecies comparisons show identities between 93.7% and 100% while all interspecies comparisons are in the range of 84.2–89.6%. Comparisons between different morphotypes of the same species are in the same range as comparisons between identical morphotypes. Since ranges of intra- and inter- species comparisons are not overlapping (barcoding gap), a simple calculation of identities between sequences will be sufficient to assign a sequence to a particular species allowing diagnosis without phylogenetic reconstruction.

### Polymorphic *Marshallagia* species

The data presented here clearly show that *M. marshalli* is a polymorphic species and that *M. occidentalis* and *M. uzbekistanica* should be considered synonyms and that their descriptions detail the morphology of rare morphotypes of *M. marshalli*. In addition, the morphotypes *M. schumakovitschi* and *M. trifida* belong to the same species. Although *M. schumakovitschi* represents the major morphotype, this name should be considered a synonym of *M. trifida* since *M. trifida* was described earlier and this name has therefore priority according to the rules of zoological nomenclature. Data from Lv et al. [44] furthermore suggest that *M. grossospiculum* is a minor morphotype of the species *M. mongolica* and only the latter represents a valid species name. However, absence of morphological data in the publication by Lv et al. [44] suggests that further confirmation is warranted.

It remains unclear whether all *Marshallagia* species are polymorphic but the new data presented here confirm certain pairs of morphotypes that must be considered to be the same species. For other species, the availability of *cox*1 sequences will allow to group more morphotypes once new sequences from morphologically identified and documented material become available.

### *Marshallagia* DNA sequences from specimens of unclear origin

The phylogenetic tree in Fig. 2 contains several GenBank entries from samples of doubtable origin. In particular, there is no available description for *M. hsui* and the name is considered to be a *nomen nudum* [2]. The sequences obviously belong to a *Marshallagia* species but without a detailed, publicly available morphological description, it is impossible to decide whether they represent an undescribed species or if they should be assigned to one of the many described species for which no *cox*1 reference sequences are currently available.

The *M. occidentalis* specimens from China for which no morphological description was provided by Lv et al. [44] have presumably been identified incorrectly. The sequences apparently represent *Marshallagia* sequences but the assignment to *M. occidentalis* is in contrast to all data summarized by Hoberg et al. [2] and to the results of the present study, which all consider *M. occidentalis* as a minor morphotype of *M. marshalli*. Without voucher material deposited in a museum, the taxonomic value of these sequences will remain very limited and only after identical/highly similar sequences will be reported with a detailed morphological description and deposited voucher material, the sequences will be of epidemiological and taxonomic value.

## Conclusions

Species limits remain poorly defined within the genus *Marshallagia* (and presumably other, particularly polymorphic Ostertagiinae as well) where subtle morphological differences, high morphological or genetic variability, incomplete descriptions, and circumscribed differential diagnoses hinder identification [2, 43, 46, 47]. It was shown in the present study that at a number of described and named taxa from Eurasia are in fact only synonyms of previously established major or minor morphotypes. For holarctic species such as *M. marshalli* with its different morphotypes, it would be highly interesting to compare Eurasian and North American specimens regarding mitochondrial genotypes to determine if there is further population genetic structuring or even different genospecies on both continents. In any case, free access to species descriptions, including approaches to overcome language barriers such as very limited accessibility of original parasitic nematode descriptions in Russian for western parasitologists and *vice versa*, would be required to improve the situation. Missing of accurate and detailed figures and difficult access to representative type-specimens is especially problematic and complicates the possibility of complete and direct comparisons among otherwise similar species and respective morphotypes [2]. Accordingly, comprehensive revision of the genus *Marshallagia* appears warranted but is currently unfortunately unrealistic.

## Supplementary information

**Additional file 1: Text S1.** Detailed morphological description of the *Marshallagia* species and morphotypes.

**Additional file 2: Figure S1.** Maximum likelihood phylogenetic tree calculated based on ITS2 sequences. *Teladorsagia circumcincta* sequences were used as the outgroup. Rapid bootstrapping and Shimodaira-Hasegawa approximate likelihood ratio test results are shown before and after the slash, respectively. M1-M14 indicate voucher designations from the present study.

## Data Availability

All data generated or analyzed during this study are included in this article and its additional files. Sequence data were deposited in the GenBank database under the accession numbers listed in Table 3.

## Abbreviations

rRNA: ribosomal RNA
ITS1: internal transcribed spacer 1
ITS2: internal transcribed spacer 2
*cox*1: cytochrome *c* oxidase subunit 1 gene

## References

[1] Ivashkin VM, Oripov AO, Sonin MD (1989). Manual for determinative helminths of sheep and goats.

[2] Hoberg EH, Abrams A, Pilitt PA, Jenkins EJ (2012). Discovery and description of a new Trichostrongyloid species (Nematoda: Ostertagiinae), abomasal parasites in mountain goat, *Oreamnos americanus*, from the western Cordillera of North America. J Parasitol..

[3] Irgashev IH (1972). Helminths and helminthiasis karakul sheep.

[4] Oripov SA. Trihostrongylidiasis of sheep in Uzbekistan and their control. Ph.D. thesis, All-Union Scientific Research Institute for Parasitology K. I. Skryabin, Russia; 1983.

[5] Abramatov MB, Amirov OO, Ruziev BKh, Kuchboev AE (2014). Helmintocenosis of abomasum from domestic ruminants of Uzbekistan. Biol Sci Kazakhstan..

[6] Kuchboev AE, Amirov OO, Karimova RR, Asakawa M (2016). Nematodes in the digestive tract of domestic ruminants in Uzbekistan. Jpn J Vet Parasitol..

[7] Drozdz J. The question of genetic isolation and of permanent coincidence of some species of the subfamily Ostertagiinae. In: Proceedings of the third international congress of parasitology. Munich; 1974. p. 477–8.

[8] Drozdz J (1979). Genetic isolation as a criterion for defining a species within the parasitic nematodes. Wiad Parazytol..

[9] Drozdz J (1995). Polymorphism in the Ostertagiinae Lopez-Neyra, 1947 and comments on the systematics of these nematodes. Syst Parasitol..

[10] Gibbons LM, Khalil LF (1982). A key for the identification of genera of the nematode family Trichostrongylidae Leiper, 1912. J Helminthol..

[11] Lichtenfels JR, Pilitt A (1989). Cuticular ridge patterns of *Marshallagia marshalli* and *Ostertagia occidentalis* (Nematoda: Trichostrongyloidea) parasitic in ruminants of North America. Proc Helminthol Soc Wash..

[12] Wyrobisz A, Kowal J, Nosal P (2016). Insight into species diversity of the Trichostrongylidae Leiper, 1912 (Nematoda: Strongylida) in ruminants. J Helminthol..

[13] Ramünke S, de Almeida Borges F, von Son-de Fernex E, von Samson-Himmelstjerna G, Krücken J (2018). Molecular marker sequences of cattle *Cooperia* species identify *Cooperia spatulata* as a morphotype of *Cooperia punctata*. PLoS One..

[14] Bredtmann CM, Krücken J, Kuzmina T, Louro M, de Madeira Carvalho LM, von Samson-Himmelstjerna G (2019). Nuclear and mitochondrial marker sequences reveal close relationship between *Coronocyclus coronatus* and a potential *Cylicostephanus calicatus* cryptic species complex. Infect Genet Evol..

[15] Zarlenga DS, Hoberg EP, Stringfellow F, Lichtenfels JR (1998). Comparisons of two polymorphic species of *Ostertagia* and phylogenetic relationships within the Ostertagiinae (Nematoda: Trichostrongyloidea) inferred from ribosomal DNA repeat and mitochondrial DNA sequences. J Parasitol..

[16] Dallas JF, Irvine RJ, Halvorsen O (2000). DNA evidence that *Ostertagia gruehneri* and *Ostertagia arctica* (Nematoda: Ostertagiinae) in reindeer from Norway and Svalbard are conspecific. Int J Parasitol..

[17] Chilton NB, Newton LA, Beveridge I, Gasser RB (2001). Evolutionary relationships of trichostrongyloid nematodes (Strongylida) inferred from ribosomal DNA sequence data. Mol Phylogenet Evol..

[18] Santin-Duran M, de la Fuente C, Alunda JM, Rosental BM, Hoberg EP (2002). Identical ITS-1 and ITS-2 sequences suggest *Spiculopteragia asymmetrica* and *Spiculopteragia quadrispiculata* (Nematoda: Trichostrongylidae) constitute morphologically distinct variants of a single species. J Parasitol..

[19] Kuznetsov DN (2009). The results of the comparative study of the spacer region of the ribosomal DNA *Teladorsagia circumcincta* and *T. trifurcata* (Nematoda: Ostertagiinae). Russian Parasitol J..

[20] Kuznetsov DN (2010). On the question of species identity *Ostertagia ostertagi* and *Ostertagia lyrata* (Nematoda: Ostertagiinae). Parazitologiya..

[21] Aksenov AP. Nematode’s subfamily Ostertagiinae Lopez-Neyra, 1947: systematics and phylogeny. Ph.D. Thesis, A. N. Severtsov Institute of Ecology and Evoltion, Russia; 2013.

[22] Amirov OO, Kuchboev AE (2013). Molecular characterization of nematodes *Teladorsagia circumcincta* and *T. trifurcata* (Trichostrongylidae: Ostertagiinae) using the spacer regions of ribosomal DNA. Bull Natl Univ Uzbekistan..

[23] Amirov OO, Kuchboev AE (2014). Molecular genetic analysis of *Ostertagia ostertagi* and *O. lyrata* (Trichostrongylidae). Bull Gullistan State Univ..

[24] Amirov OO, Mirzaeva GC, Kuchboev AE (2014). Molecular genetic analysis species of *Teladorsagia sircumcincta* and *Teladorsagia davtiani* (Nematoda: Ostertagiinae). Infect Immunol Pharmacol..

[25] Dallas JF, Irvine RJ, Halvorsen O (2001). DNA evidence that Marshallagia marshalli Ransom, 1907 and *M. occidentalis* Ransom, 1907 (Nematoda: Ostertagiinae) from Svalbard reindeer are conspecific. Int J Parasitol..

[26] Kuznetsov DN (2001). Taxonomic revision of the genus *Orloffia* (Nematoda: Ostertagiinae) based on an ITS-2 rDNA study. Biol Bull..

[27] Blouin MS (2002). Molecular prospecting for cryptic species of nematodes: mitochondrial DNA *versus* internal transcribed spacer. Int J Parasitol..

[28] Gasser RB, Chilton NB, Hoste H, Beveridgi L (1993). Rapid sequencing of rDNA from single worms and eggs of parasitic helminthes. Nucleic Acids Res..

[29] Duscher GG, Harl J, Fuehrer HP (2015). Evidence of *Troglotrema acutum* and *Skrjabingylus* sp. coinfection in a polecat from lower Austria. Helminthologia..

[30] Katoh K, Standley DM (2013). MAFFT multiple sequence alignment software version 7: improvements in performance and usability. Mol Biol Evol..

[31] Taly JF, Magis C, Bussotti G, Chang JM, Di Tommaso P, Erb I (2011). Using the T-Coffee package to build multiple sequence alignments of protein, RNA, DNA sequences and 3D structures. Nat Protoc..

[32] Popescu AA, Huber KT, Paradis E (2012). ape 3.0: new tools for distance-based phylogenetic and evolutionary analysis in R. Bioinformatics..

[33] R Development Core Team. R: A language and environment for statistical computing. R Foundation for Statistical Computing. Vienna: R Foundation for Statistical Computing; 2020. https://www.R-project.org.

[34] Pohlert T. The pairwise multiple comparison of mean ranks package (PMCMR). R package. 2014. http://CRAN.R-project.org/package=PMCMR.

[35] Xia X, Lemey P. editors. 209. Assessing substitution saturation with DAMBE. In: Lemey P, Salemi M., Vandamme A-M, editors. The Phylogenetic Handbook: A Practical Approach to Phylogenetic Analysis and Hypothesis Testing. 2nd ed. Cambridge: Cambridge University Press; 2009. p. 615–30.

[36] Xia X (2013). DAMBE5: a comprehensive software package for data analysis in molecular biology and evolution. Mol Biol Evol..

[37] Kalyaanamoorthy S, Minh BQ, Wong TKF, von Haeseler A, Jermiin LS (2017). ModelFinder: fast model selection for accurate phylogenetic estimates. Nat Methods..

[38] Hoang DT, Vinh LS, Chernomor O, Minh BQ, von Haeseler A (2017). UFBoot2: improving the ultrafast bootstrap approximation. Mol Biol Evol..

[39] Guindon S, Dufayard JF, Lefort V, Anisimova M, Hordijk W, Gascuel O (2010). New algorithms and methods to estimate maximum-likelihood phylogenies: assessing the performance of PhyML 3.0. Syst Biol..

[40] Ransom BH. Notes on parasitic nematodes, including descriptions of new genera and species, and observations of life histories. U.S. Department of Agriculture, Bureau of Animal Industry Circular 116; 1907.

[41] Ransom BH. The nematodes parasitic in the alimentary tract of cattle, sheep and other ruminants. Bureau of Animal Industry, U.S. Department of Agriculture, U.S. Government Printing Office, Washington, D.C., Bulletin 127; 1911.

[42] Azimov DA, Dadaev D (2001). *Marshallagia uzbekistanica* sp. n., a new nematode of sheep and goats. Uzbek Biol J..

[43] Asadov SM (1960). Helminthofauna ruminants USSR and its ecological and geographical analysis.

[44] Lv J, Zhang Y, Feng C, Yuan X, Sun D, Deng J (2016). Species discrimination in the subfamily Ostertagiinae of northern China: assessment of DNA barcode in a taxonomically challenging group. Parasitol Res..

[45] Sun MM, Han L, Zhang FK, Zhou DH, Wang SQ, Ma J (2018). Characterization of the complete mitochondrial genome of *Marshallagia marshalli* and phylogenetic implications for the superfamily Trichostrongyloidea. Parasitol Res..

[46] Hu J, Jiang Y (1984). *Marshallagia brevicauda* new species (Nematoda, Trichostrongylidae) from sheep. Acta Veterinaria et Zootechnica Sin..

[47] Luo JZ, Zhang HJ, Wu BS, Bai ZY (1993). Two new species of *Marshallagia* from Qinghai, China. Acta Zootaxonomica Sin..

