# Supplementary material for: Molecular analysis of polymorphic species of the genus Marshallagia (Nematoda: Ostertagiinae)
Source: Parasit Vectors. 2020 Aug 12;13:411. doi: 10.1186/s13071-020-04265-1 (PMC7425555; doi:10.1186/s13071-020-04265-1)
Supplement: Supplementary file 1 — Additional file 1: Text S1. Detailed morphological description of the Marshallagia species and morphotypes. [file 13071_2020_4265_MOESM1_ESM.pdf]

Additional file 1: Text S1

Morphological description of the *Marshallagia* species

Representatives of the genus *Marshallagia* significantly differ from other Ostertagiinae by their relatively powerful bursa with large lateral lobes. According to our data, the species of *Marshallagia marshalli* Ransom, 1907 is widespread in Uzbekistan and in terms of prevalence it ranks on the first place among nematodes of the genus. Thus, in the investigated small ruminants, it was found in 55.3% of the individual hosts [5, 6]. Drozdz [7, 8] described a phenomenon of a regular co-occurrence of these rather rare (minor) species in pairs with the more numerous (major) species. In each pair, a significant correlation between the intensity of infection of major and minor species is usually observed. Based on this, the analyzed samples of *Marshallagia* were sorted into three groups containing such co-existing pairs of morphotypes (Fig.1). The description of these species and morphotypes of nematodes is provided below.

1. ***Marshallagia marshalli*** Ransom, 1907 (Fig. 1a)

Hosts: *Ovis aries*

Localization: abomasum

Location: obtained during the necropsy of sheep in Kashkadarya region, Uzbekistan.

Male (number of specimens in the original material collected by Abramatov et al. [5] and Kuchboev et al. [6]: n=26, Table 4)). Mean body length was 10.50 mm. The mean length of the bursa was 360 µm. The copulatory bursa is symmetrical, elongate, strongly bilobed, lacking a prominent dorsal lobe. The bursa represents the typical structure of major morphotypes. The mean length of the dorsal ray was 370 µm. It is split (bifurcated) at some distance from the tip, after which it is split once more just a few microns from the tip. Spicules are 250 µm long and they are light yellow. In the posterior quarter the spicule splits into three processes, the lateral of which has a pointed end and is provided with a vesicular membrane. The two other processes of spicules have almost equal length. Of these, the ventral is very thin, with a slight

swelling at the tip; and the dorsal process is more massive with a mushroom-shaped outgrowth at the end. A Gubernaculum is absent.

We defined this specimen as *Marshallagia marshalli* Ransom, 1907. The observed structure of the spicules shows a large similarity with the paratype specimens of the species (see Table 1, CHM 14768) having a thin ventral process of the spicule, which is close in length to the middle process. The lateral process of the spicule exceeds in length the two other processes.

2. ***Marshallagia occidentalis*** Ransom, 1907 (Fig. 1d)

Hosts: *Ovis aries*

Localization: abomasum

Location: obtained during the necropsy of a sheep in the Kashkadarya region.

Male (number of specimens in the original material collected by Abramatov et al. [5] and Kuchboev et al. [6]: n=22, Table 4)). Mean body length was 13.30 mm. The mean length of the bursa was 366 µm. The length of dorsal ray is 241 µm. The dorsal ray of the bursa is split approximately at the level of the distal third.

Spicules are powerful, the mean length was 280 µm. Near the middle of their length, the spicules are divided into three processes: two ventral and one dorsal. The external ventral process is longer than the internal process, giving the impression as if it was obliquely cut at the end; the internal process is short and thin, with a pointed end. A clearly visible gubernaculum is present; its diameter is greatly reduced at the posterior part.

This specimen was identified as *M. occidentalis* Ransom, 1907. The presence of the gubernaculum and the characteristic types of spicules were essential for this identification.

3. ***Marshallagia schumakovitschi*** Kadyrov, 1959 (Fig.1b)

Hosts: *Ovis aries*

Localization: abomasum.

Location: obtained from a sheep in the Bukhara region, Uzbekistan

Male (number of specimens in the original material collected by Abramatov et al. [5] and Kuchboev et al. [6]: n=17, Table 4)). The mean length of the body was 11.90 mm. The mean length of the bursa was 357 µm. The mean length of the dorsal ray was 300 µm. The symmetrical dorsal ray bifurcates at an acute angle, with each branch first passing along the lateral process, and then terminally divided into two branches, of which the lateral one is longer than the median one.

Mean length of the spicules was 240 µm. In the sixth part of its length, the spicule distally splits into three processes. The lateral process is bent hook-shaped in a median aspect and covered with a transparent membrane, noticeable when the spicules are separated from the bursa. The medio-ventral process is shorter than the lateral one and is the thinnest of all. A gubernaculum is absent.

This specimen was identified as *Marshallagia schumakovitschi* Kadyrov, 1959. The basis for this determination was the presence on the lateral process of the spicules with a hook-shaped end directed towards the median line.

4. ***Marshallagia trifida*** Guille, Marotel et Penisset, 1911 (Fig.1e)

Hosts: *Ovis aries*

Localization: abomasum

Location: obtained from a sheep in the Bukhara region.

Male (number of specimens in the original material collected by Abramatov et al. [5] and Kuchboev et al. [6]: n=18, Table 4)). The mean body length was 13.96 mm. The mean length of the bursa was 709 µm. The dorsal ray had a mean length of 259 µm. The thickness of the bursal rays is ordered as follows: external-lateral - almost the same width as latero-ventral and median-lateral, then in descending order follow: posterior lateral, external-dorsal and external-ventral.

Spicules were on average 295 µm long. Slightly after the middle, the spicules are divided into three branches: the interior – the shortest and narrowest, pointed at the end

– and the two outer ones, overlapping one another with trough-shaped bends at the ends. The external dorsal appendage of the spicules is the most massive. The gubernaculum is spindle shaped. The proximal end of it is split into two short processes.

This specimen was defined as minor species *Marshallagia trifida* Guille, Marotel et Penisset, 1911.

5. ***Marshallagia sogdiana*** (Pulatov, 1985) (Fig. 1f)

Hosts: *Ovis aries*

Localization: abomasum

Location: obtained from a sheep in the Bukhara region.

Male (number of specimens in the original material collected by Abramatov et al. [5] and Kuchboev et al. [6]: n=12, Table 4)). The mean body length was 13.55 mm. The mean lengths of the bursa was 745 µm. The mean lengths of the dorsal ray was 253 µm. The two lobed bursa is equipped with two bursal membranes: dorsal and accessory. The bursal rays are of unequal thickness: the most powerful is the poster ventral antero-lateral ray followed by medio-lateral and postero-lateral rays.

Spicules are 284 µm in length on average. The proximal ends of the spicules show a peculiar, disc-like structure. In the middle, the spicules are divided into three processes: two ventral and one dorsal. The distal spicules have a membrane in the form of a sheath. A gubernaculum is present.

This specimen was defined as *Marshallagia sogdiana* (Pulatov, 1985).

6. ***Marshallagia uzbekistanica*** Azimov and Dadaev, 2001 (Fig. 1c)

Hosts: *Ovis aries*

Localization: abomasum

Location: obtained from a sheep in the Bukhara region.

Male (number of specimens in the original material collected by Abramatov et al. [5]: n=5, Table 4)). The mean length of the body was 12.40 mm. The mean length of the bursa was 366 µm. The length of the dorsal ray was 241 µm on average. The dorsal ray from the base bifurcates, each branch in turn is separated by the lateral branch and at the end the dorsal ray splits into two processes.

The mean length of the spicules was 120 µm. Spicule length of the same individual was unequal, building a peculiar structure. The spicules on the proximal end are slightly chitinized with a granular structure. In the distal third of the length of the spicule it is split into three processes. A gubernaculum is absent.

This specimen was identified as *M. uzbekistanica* Azimov and Dadaev, 2001. The characteristic for identification was a very peculiar structure and small size of the spicules.

Thus, on the basis of morphological characteristics and attributes, we identified six morphotypes of *Marshallagia*: *M. marshalli, M. occidentalis, M. schumakovitschi, M. trifida, M.sogdiana* and *M. uzbekistanica.*
