# Supplementary material for: Molecular analysis of polymorphic species of the genus Marshallagia (Nematoda: Ostertagiinae)
Source: Parasit Vectors. 2020 Aug 12;13:411. doi: 10.1186/s13071-020-04265-1 (PMC7425555; doi:10.1186/s13071-020-04265-1)

Additional file 2: Figure S1. Maximum likelihood phylogenetic tree calculated based on ITS2 sequences. *Teladorsagia circumcincta* sequences were used as outgroup. Rapid bootstrapping and Shimodaira-Hasegawa approximate likelihood ratio test results are shown before and after the slash, respectively. M1-M14 indicate voucher designations from the present study.

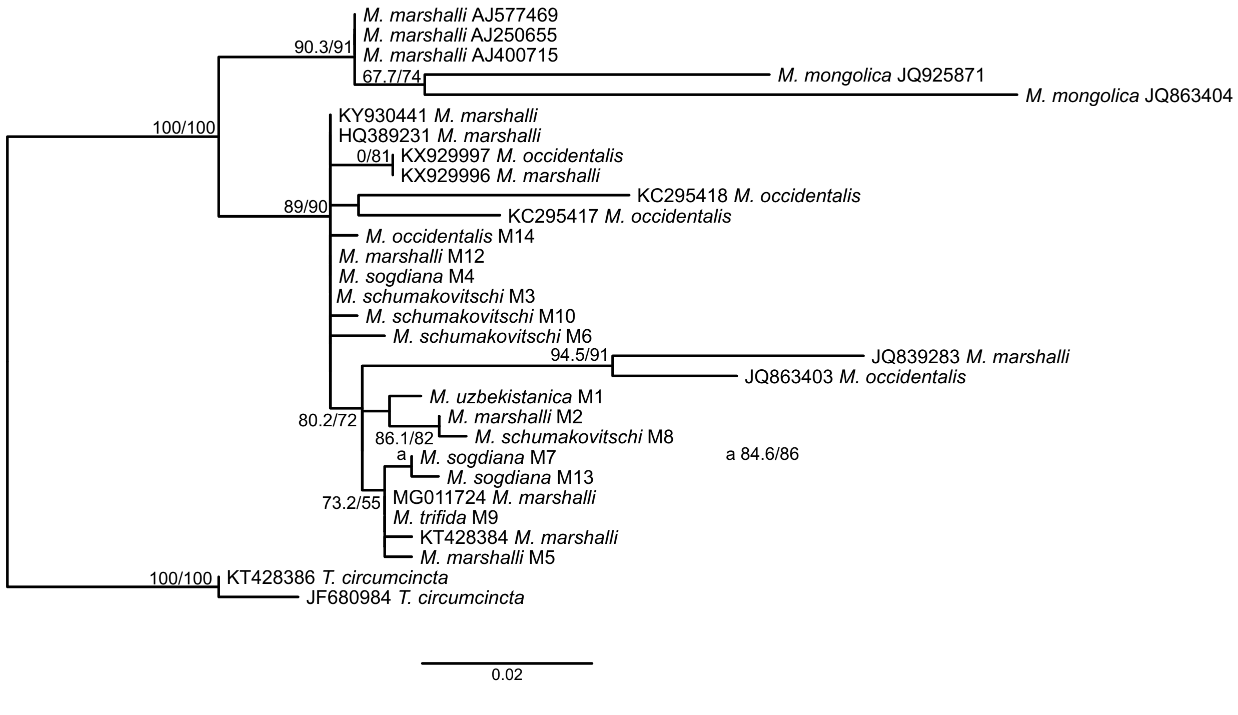

Supplement: Supplementary file 2 — Additional file 2: Figure S1. Maximum likelihood phylogenetic tree calculated based on ITS2 sequences. Teladorsagia circumcincta sequences were used as the outgroup. Rapid bootstrapping and Shimodaira-Hasegawa approximate likelihood ratio test results are shown before and after the slash, respectively. M1-M14 indicate voucher designations from the present study. [file 13071_2020_4265_MOESM2_ESM.pdf]
